# Human Cord Blood Stem Cell-Modulated Regulatory T Lymphocytes Reverse the Autoimmune-Caused Type 1 Diabetes in Nonobese Diabetic (NOD) Mice

**DOI:** 10.1371/journal.pone.0004226

**Published:** 2009-01-19

**Authors:** Yong Zhao, Brian Lin, Robert Darflinger, Yongkang Zhang, Mark J. Holterman, Randal A. Skidgel

**Affiliations:** 1 Department of Medicine, University of Illinois at Chicago, Chicago, Illinois, United States of America; 2 Department of Pharmacology, University of Illinois at Chicago, Chicago, Illinois, United States of America; 3 Department of Surgery, University of Illinois at Chicago, Chicago, Illinois, United States of America; New York University School of Medicine, United States of America

## Abstract

**Background:**

The deficit of pancreatic islet β cells caused by autoimmune destruction is a crucial issue in type 1 diabetes (T1D). It is essential to fundamentally control the autoimmunity for treatment of T1D. Regulatory T cells (Tregs) play a pivotal role in maintaining self-tolerance through their inhibitory impact on autoreactive effector T cells. An abnormality of Tregs is associated with initiation of progression of T1D.

**Methodology/Principal Findings:**

Here, we report that treatment of established autoimmune-caused diabetes in NOD mice with purified autologous CD4^+^CD62L^+^ Tregs co-cultured with human cord blood stem cells (CB-SC) can eliminate hyperglycemia, promote islet β-cell regeneration to increase β-cell mass and insulin production, and reconstitute islet architecture. Correspondingly, treatment with CB-SC-modulated CD4^+^CD62L^+^ Tregs (mCD4CD62L Tregs) resulted in a marked reduction of insulitis, restored Th1/Th2 cytokine balance in blood, and induced apoptosis of infiltrated leukocytes in pancreatic islets.

**Conclusions/Significance:**

These data demonstrate that treatment with mCD4CD62L Tregs can reverse overt diabetes, providing a novel strategy for the treatment of type 1 diabetes as well as other autoimmune diseases.

## Introduction

Current stem cell-based therapy, along with islet transplantation and promotion of β-cell regeneration by drugs and reprogramming of adult pancreatic exocrine cells to β cells, are promising approaches to treat type 1 diabetes (T1D) [1]–[7]. However, autoreactive effector T cells may destroy these newly-generated insulin-producing cells, thereby minimizing their therapeutic potential. A major challenge for treatment of T1D is to identify therapeutic approaches that fundamentally modulate autoimmune responses. Regulatory T cells (Tregs) play a crucial role in maintaining homeostasis and self-tolerance through their inhibitory impact on autoreactive effector T cells [8]–[14], such as releasing immunosuppressive cytokines interleukin-10 (IL-10) and/or transforming growth factor-β (TGF-β). Although defects of effector T cells [15]–[18] or antigen-presenting cells [19]–[23] could play a role, increasing evidence demonstrates that abnormalities of Tregs, either in cell number [24]–[26] or in function [10], [27]–[32], are associated with initiation and progression of T1D, both in diabetic patients and animal models. Thus, the manipulation of Tregs for treatment of T1D is an attractive approach. Nevertheless, only a limited number of studies have focused on restoration of impaired Treg function to confer protection against autoimmune diabetes. We identified a novel type of stem cells from human umbilical cord blood, designated cord blood stem cells (CB-SC). Recently, we found that CB-SC displayed immuno-modulatory effects on human allogeneic T lymphocytes via *in vitro* co-culture with stem cells [3], [33], the same as mesenchymal stem cells derived from bone marrow [34]. Based on this work, we hypothesized that CB-SC might also modulate Treg function in autoimmune-caused T1D. Here, we report that CB-SC can correct functional defects of mouse CD4^+^CD62L^+^ Tregs, leading to reversal of overt diabetes in an autoimmune-caused diabetic NOD mouse model. These findings may have implications for the development of novel human therapies.

## Results

### CB-SC modulation of NOD mouse regulatory T lymphocytes

**Figure 1:**
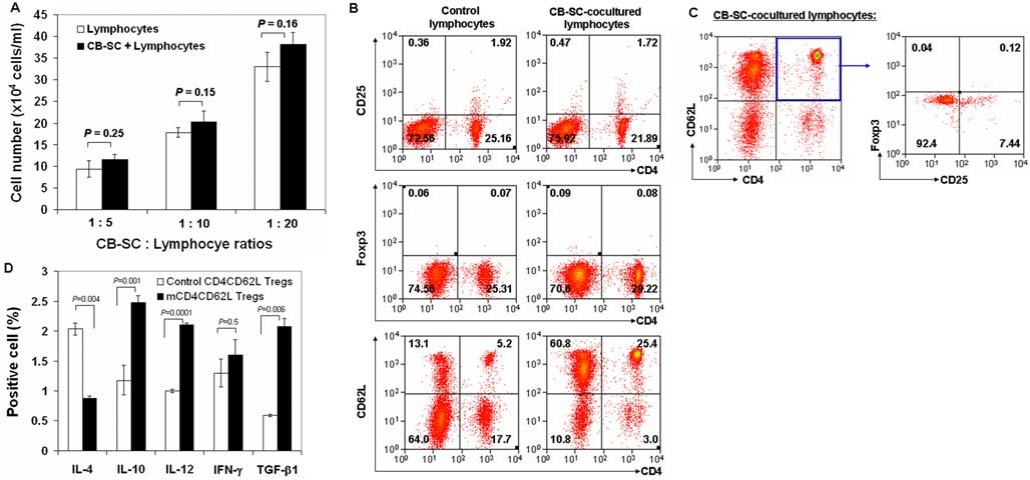
Flow analysis of CD4^+^CD62L^+^ Tregs following i*n vitro* co-culture with human cord blood stem cells CB-SC. Mouse spleen lymphocytes were isolated from female NOD mice (aged 6–8 weeks) and then co-cultured with CB-SC for 2–4 days at different ratios (A) or a ratio 1∶10 of CB-SC∶lymphocytes (B–D). Subsequently, the total lymphocytes were harvested for following cell count or flow analyses. Lymphocytes cultured in absence of CB-SC served as control. (A) CB-SC have no significant effect on mouse lymphocyte proliferation during co-culture at different ratios of CB-SC∶lymphocytes. Data represent mean±s.d. of four experiments. (B) Percentage of CD4^+^CD25^+^ Treg, CD4^+^Foxp3^+^ Treg, and CD4^+^CD62L^+^ Treg after *in vitro* co-culture with CB-SC. (C) Flow analysis of CD25 and Foxp3 expressions in CD4^+^CD62L^+^ Tregs after *in vitro* co-culture with CB-SC. (D) Flow analysis on CD4^+^CD62L^+^ Tregs after intra-cellular cytokine staining. Isotype-matched IgG served as control. Data in B–C are representative of three to five experiments.

To investigate the therapeutic potential of Tregs in T1D, we employed an experimental nonobese diabetic (NOD) mouse model. Initially, we tested the co-culture of CB-SC and NOD mouse spleen-derived lymphocytes and found that co-culture with CB-SC did not significantly stimulate the proliferation of mouse lymphocytes at different ratios of CB-SC∶lymphocytes (1∶5, 1∶10, and 1∶20) (**Figure 1A**, *p* = 0.25, *p* = 0.15, *p* = 0.16 respectively), which is similar to the co-culture of CB-SC and human lymphocytes [33]. Next, we analyzed co-cultures of CB-SC and mouse lymphocytes for the presence of Tregs including conventional CD4^+^CD25^+^ Treg and CD4^+^Foxp3^+^ Treg, and the CD4^+^CD62L^+^ Treg. We found no significant differences in CD4^+^CD25^+^ Treg and CD4^+^Foxp3^+^ Treg in total mouse spleen lymphocytes that were either cultured alone or with CB-SC. In contrast, the percentage of CD4^+^CD62L^+^ Treg was increased about 5-fold after co-culture with CB-SC (**Figure 1B**). Further flow cytometry revealed that only a very small proportion of these CD4^+^CD62L^+^ Tregs was CD4^+^CD25^+^CD62L^+^Foxp3^+^ positive (**Figure 1C**), and this percentage was not different between lymphocytes co-cultured with or without CB-SC (0.11±0.04% vs 0.10±0.03%, *P* = 0.44). We subsequently focused on CD4^+^CD62L^+^ Tregs, which were primarily affected by co-culture with CB-SC (designated CB-SC-modulated CD4^+^CD62L^+^ Tregs, mCD4CD62L Tregs).

To document modulation of CD4^+^CD62L^+^ Tregs by CB-SC after *in vitro* co-culture, intracellular cytokines related to helper T (Th)1 and Th2 immune responses were measured using flow analysis (**Figure 1D**). Results demonstrated that the IL-4 level was significantly down-regulated (*p* = 0.004), whereas IL-10, IL-12 and TGF-β1 levels were up-regulated in mCD4CD62L Tregs compared with control CD4CD62L Tregs (*p* = 0.001, *p* = 0.0001, and *p* = 0.006 respectively). In contrast, the IFN-γ expression did not change following co-culture with CB-SC (**Figure 1D**, *p* = 0.5). Next, we investigated expression of Th1-Th2-Th3 cell-related genes by using quantitative real time PCR array in the purified CD4^+^CD62L^+^ Tregs following co-culture with CB-SC. Results demonstrated that mCD4CD62L Tregs displayed marked down-regulation of Th cell-related genes including multiple cytokines and their receptors, chemokines and their receptors, cell surface molecules, along with signaling pathway molecules and transcription factors (**Table S1**). These data clearly indicate that *in vitro* co-culture with CB-SC causes substantial modifications of gene expression in mouse CD4^+^CD62L^+^ Tregs, specifically for function-related cytokine and chemokine genes.

### CB-SC-modulated CD4^+^CD62L^+^ Tregs (mCD4CD62L Tregs) correct hyperglycemia in overt diabetic NOD mice

**Figure 2:**
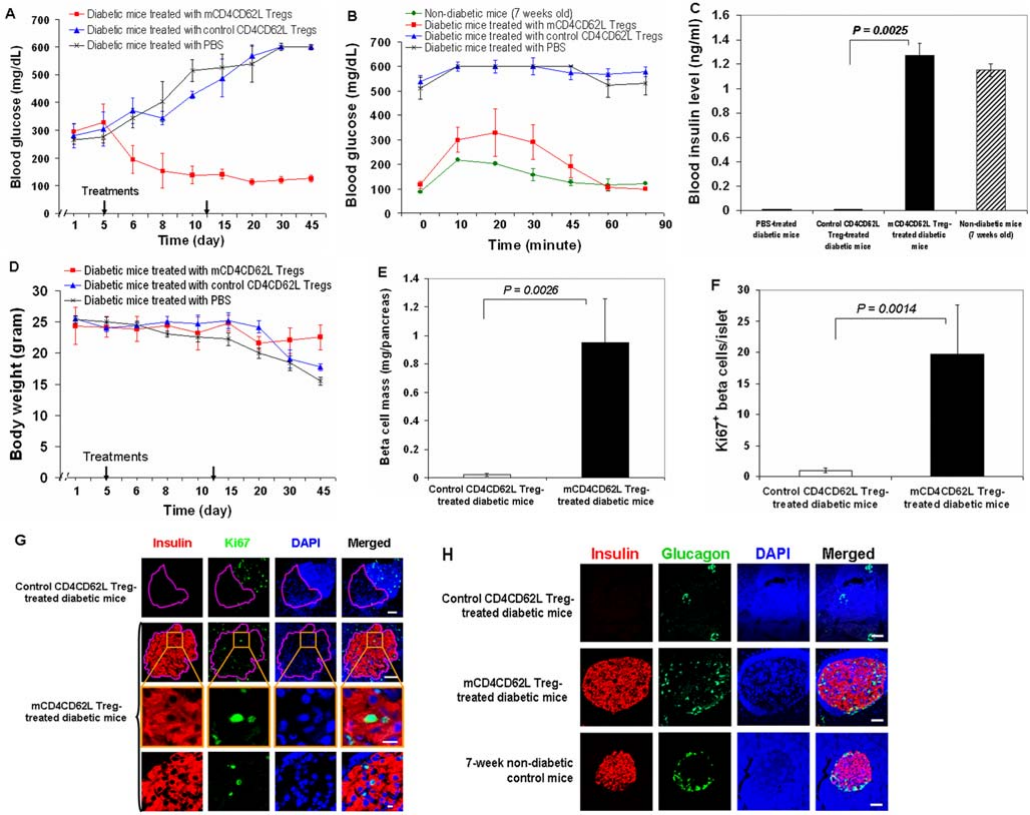
CB-SC-modulated CD4^+^CD62L^+^ Tregs (mCD4CD62L Tregs) reverse hyperglycemia in overt diabetic NOD mice. Mouse spleen lymphocytes isolated from female NOD mice (aged 6–8 weeks) were co-cultured with CB-SC for cell sorting as described in methods. These purified CD4^+^CD62L^+^ Tregs (mCD4CD62L Tregs) were used for treatment of spontaneously-developed autoimmune-caused diabetes in NOD mice. The purified CD4^+^CD62L^+^ Tregs from lymphocytes not co-cultured with CB-SC (control CD4CD62L Tregs) served as control for mCD4CD62L Tregs. Injection of vehicle PBS served as an additional control (n = 5). (A) The mCD4CD62L Tregs correct hyperglycemia in diabetic NOD mice. Overt diabetic NOD mice were treated with mCD4CD62L Tregs (total 5 million cells/mouse, i.p., red line; representative data are from 6 diabetic mice sensitive to mCD4CD62L Treg treatment with euglycemia, n = 8 mice). Purified control CD4CD62L Tregs served as control (total 5 million cells/mouse, i.p., blue line, n = 5 mice). PBS served as an additional control (black line, n = 5 mice). (B) Intraperitoneal glucose tolerance testing (IPGTT) 3 weeks following the 1^st^ treatment with mCD4CD62L Tregs. Seven-week old NOD mice served as normal control. (C) Determination of blood insulin levels by ELISA. (D) Effect of treatment on mouse body weight. (E–H) Pancreatic histology analyses: Pancreata from mCD4CD62L Treg-treated diabetic mice and control mice were collected for immunohistochemistry after observation for 45 days. Representative sections are shown in each panel (G, H). (E) Morphometric analysis of pancreatic β-cell mass. Pancreatic β-cell mass was determined by point-counting morphometry on insulin-positive islet β cells followed by immunostaining with guinea pig anti-insulin Ab (Dako) and counter-staining with hematoxylin. (F) Quantification of Ki67-positive cells in pancreatic islets after double immunostaining with Ki67 and insulin Abs. Isotype-matched rabbit IgG served as control for rabbit anti-Ki67 mAb. (G) Confocal microscopy shows double-immunostaining for insulin (red) and a cell proliferation nuclear marker Ki67 (green) (scale bar, 50 µm), with a high magnification (two bottom rows, scale bar 10 µm). Control CD4CD62L Treg-treated diabetic mice (top panels) showed the Ki67-positive cells distributed in the infiltrated inflammatory cells (blue, with high density), not in β-cell area (dashed pink circle), with almost complete disappearance of β cells (red). (H) Double-immunostaining for β-cell marker insulin (red) and α-cell marker glucagon (green), followed by nuclear counter-staining with DAPI (blue). Seven-week old NOD mice served as non-diabetic control to show normal islet architecture. Scale bar, 50 µm.

Next, overt diabetic NOD mice (female, at 24–28 weeks of age) were treated with mCD4CD62L Tregs (total 5 million cells/mouse, i.p., n = 8 mice) for 5–20 days after the diagnosis of T1D to determine their therapeutic potential. The control CD4CD62L Tregs at the same cell amount (i.p., n = 5 mice) and vehicle PBS (total 200 µl/mouse, i.p., n = 5 mice) served as controls. Notably, we found that treatment with mCD4CD62L Tregs restored euglycemia in these overt diabetic mice (6/8 mice) (**Figure 2A**). However, treatment with control CD4CD62L Tregs or PBS failed to reduce hyperglycemia in diabetic mice (5/5, 5/5 mice respectively) (**Figure 2A**). Diabetic mice that had been rendered euglycemic after treatment with mCD4CD62L Tregs also showed an improved glucose tolerance test (IPGTT), similar to that of non-diabetic NOD mice at 7 weeks (**Figure 2B**). However, diabetic mice treated with PBS or control CD4CD62L Tregs maintained high glucose levels (>500 mg/dL) without any observable down-regulation (**Figure 2B**). Moreover, we monitored blood insulin levels 6 weeks after treatment with mCD4CD62L Tregs. Results showed that insulin in diabetic mice treated with control CD4CD62L Tregs or PBS vehicle was undetectable by ELISA (0.019 ng/ml sensitivity for the ELISA kit, **Figure 2C**). These mice had to be sacrificed because of severe hyperglycemia (BG>600 mg/dL) and loss of body weight (>20%) according to the protocol approved by the Animal Care Committee (**Figure 2D**). In contrast, blood insulin levels in diabetic NOD mice treated with mCD4CD62L Tregs were significantly increased (**Figure 2C**, *p* = 0.0025).

At 45 days after treatment, we subjected pancreata to histological analysis and evaluated total β-cell mass followed by immunostaining with insulin Ab on serial pancreatic sections. Morphometric analysis demonstrated that treatment with mCD4CD62L Tregs significantly increased total β-cell mass (**Figure 2E**, *p* = 0.0026). In contrast, β-cell mass was markedly lower after vehicle PBS treatment or control CD4CD62L Treg treatment (**Figure 2E**). To understand the mechanism of the increase in total β-cell mass, we determined the expression of a cell proliferation nuclear marker Ki67 [35] in pancreatic islets. Double immunostaining with insulin and Ki67 Abs revealed that 20±8 β cells/islet expressed Ki67 in pancreatic islets of mCD4CD62L Treg-treated mice (**Figure 2F and G**), which was much higher than that in pancreatic islets of mice treated with control CD4CD62L Tregs (1±0.4) (*p* = 0.0014). It suggests that *de novo* proliferation of β cells accounts for the noted increase in total β cell mass. Moreover, double immunostaining with β-cell-marker insulin and α-cell-marker glucagon revealed that pancreatic islets in diabetic mice treated with mCD4CD62L Tregs displayed a similar pattern of α- and β-cell distribution as that noted in normal islets of non-diabetic NOD mice (**Figure 2H**). However, islet architecture was completely destroyed with almost complete disappearance of β cells in the diabetic mice treated with control CD4CD62L Tregs (**Figure 2H**). Thus, treatment with mCD4CD62L Tregs can correct hyperglycemia of T1D mice by promoting β-cell regeneration and reconstitution of islet cell architecture.

### Treatment with mCD4CD62L Tregs reverses insulitis and immune dysfunction in NOD mice

**Figure 3:**
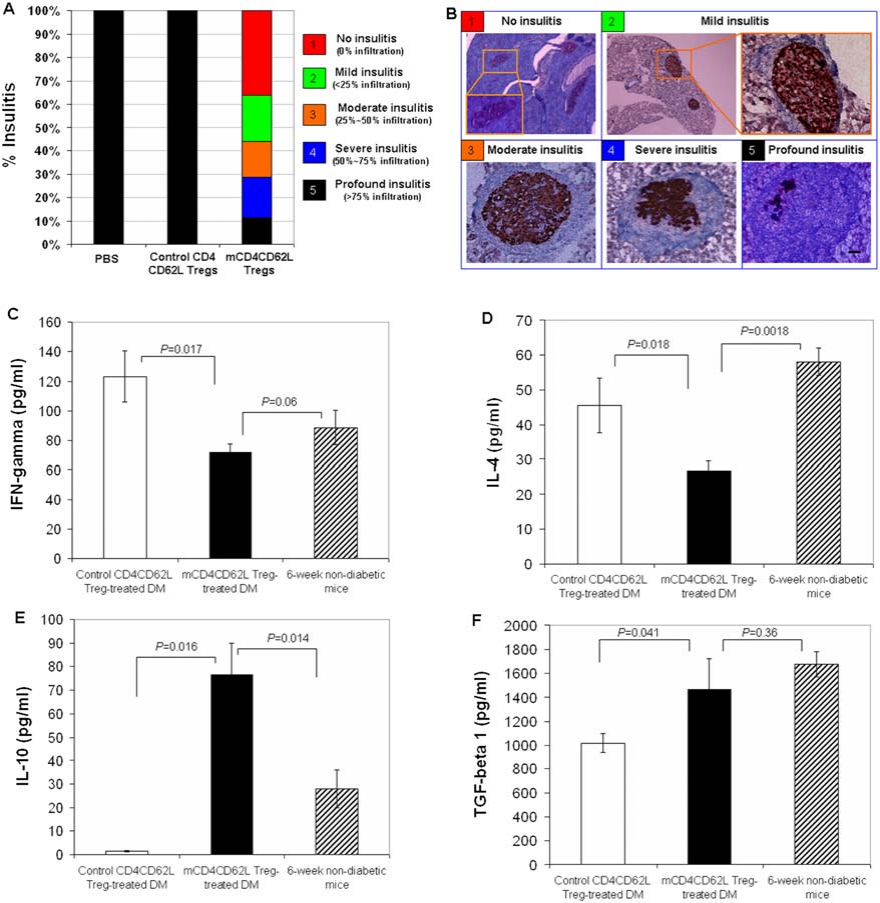
Treatment with mCD4CD62L Tregs reverses insulitis and immune dysfunction in diabetic NOD mice. Overt diabetic NOD mice treated with mCD4CD62L Tregs were sacrificed for pancreatic histological analysis and evaluation of blood cytokine levels after observation for 45 days (n = 8). The control CD4CD62L Treg-treated diabetic mice (n = 5) and PBS-treated diabetic mice (n = 5) served as controls. (A and B) Treatment with mCD4CD62L Tregs corrects insulitis in overt type 1 diabetic NOD mice. Representative data are from 6 diabetic mice (6/8 mice) sensitive to mCD4CD62L Treg treatment with euglycemia. (A) Scoring of insulitis. Pancreatic islets were scored for % mononuclear cell infiltration after immunostaining for insulin and counter-staining with hematoxylin as described in methods. (B) Representative images for different type of insulitis. Data were collected from mCD4CD62L Treg-treated diabetic NOD mice. Scale bar, 50 µm. (C) Determination of plasma IFN-γ level by ELISA. Non-diabetic NOD mice at age of 6 weeks served as normal control. (D) Measurement of plasma IL-4 level by ELISA. (E) Determination of plasma IL-10 level measured by ELISA. (F) Determination of plasma TGF-β1 level measured by ELISA. Data are shown as mean±s.d. of mouse plasma cytokine levels from three experiments.

To establish whether mCD4CD62L Tregs exert an immunosuppressive influence on autoreactive effector T cells, we performed pancreatic histological analysis and scored insulitis at 45 days after treatment. Histological evaluations showed that approximately 80% of islet β cells (profound insulitis) were destroyed in diabetic NOD mice prior to treatment. Six weeks post treatment, we found that in diabetic mice receiving mCD4CD62L Tregs, 36% of islets had no or few signs of infiltration of inflammatory cells; 20% of islets displayed mild insulitis; 15% of islets exhibited moderate insulitis; 18% of islets had severe insulitis and only 11% of islets showed profound insulitis (**Figure 3A and B**). The insulitis-free islets were of smaller size and positive for the proliferation marker Ki67 (data not shown), suggesting that these islets may have been newly generated. In contrast, all pancreatic islets in diabetic mice receiving control CD4CD62L Tregs showed massive infiltration of inflammatory cells and severe destruction of pancreatic architecture (**Figure 3A**), and had few or no insulin-positive cells present. Similarly, pancreatic histological examination demonstrated that those two mice (2/8 mice) that were resistant to mCD4CD62L Tregs treatment also displayed profound insulitis (data not shown) after 45 days observation.

To understand the molecular mechanism underlying reduction of insulitis, we measured plasma Th1/Th2 cytokine levels by ELISA. We found that Th1 cytokine IFN-γ and Th2 cytokine IL-4 were considerably reduced in the plasma of mCD4CD62L Treg-treated diabetic mice relative to control CD4CD62L Treg-treated diabetic mice (*P* = 0.017, **Figure 3C**; *P* = 0.018, **Figure 3D** respectively). In contrast, diabetic mice receiving mCD4CD62L Tregs showed a marked increase in plasma IL-10 level compared with those treated with control CD4CD62L Tregs (*P* = 0.016; **Figure 3E**) and non-diabetic NOD mice at age of 6 weeks (*P* = 0.014; **Figure 3E**). Additionally, plasma TGF-β1 level was significantly elevated in mCD4CD62L Treg-treated diabetic mice compared with control CD4CD62L Treg-treated diabetic mice (*P* = 0.041; **Figure 3F**). These data suggest that both IL-10 and TGF-β1 may contribute to an induction of immune tolerance after treatment with mCD4CD62L Tregs [36]–[38]. These data demonstrate that exposure to CB-SC induced profound changes in mCD4CD62L Tregs that helped restore “normal” islet architecture and β-cell function resulting in the suppression of diabetes.

**Figure 4:**
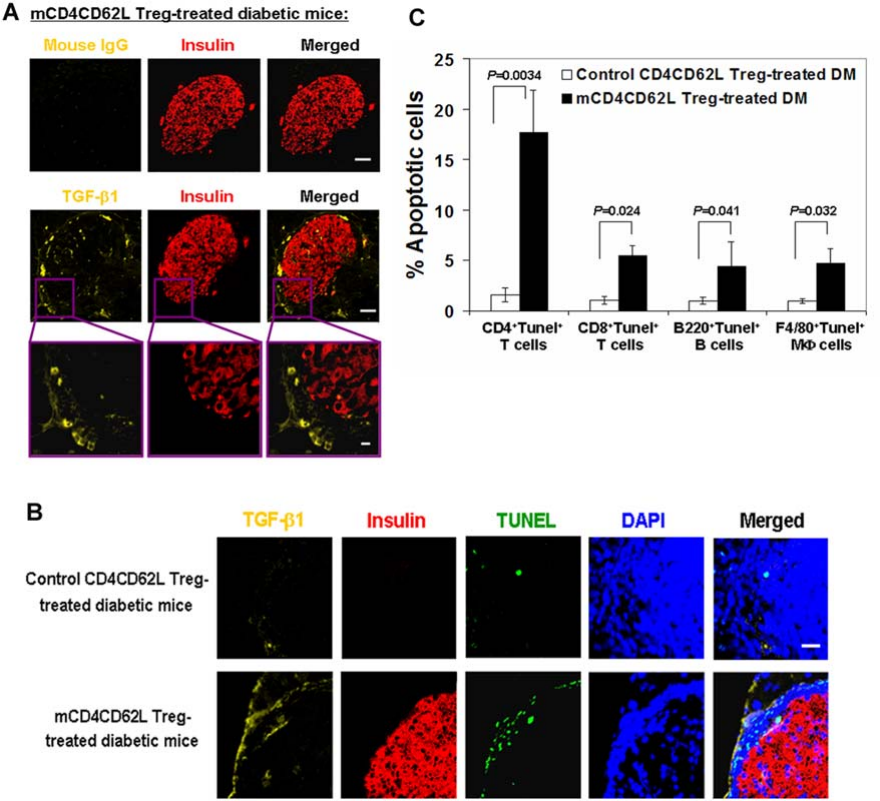
Treatment with mCD4CD62L Tregs enhance expression of TGF-β1 in pancreatic islets. Overt diabetic NOD mice treated with mCD4CD62L Tregs were sacrificed for pancreatic immunohistochemistry studies after observation for 45 days (n = 8). Representative data are from 6 diabetic mice (6/8 mice) sensitive to mCD4CD62L Treg treatment with euglycemia. The control CD4CD62L Treg-treated diabetic mice served as control (n = 5). (A) TGF-β1 staining surrounds a pancreatic islet of mCD4CD62L Treg-treated diabetic mice, as determined by double-immunostaining for TGF-β1 (yellow) and insulin (red). TGF-β1 positive cells (bright yellow) and released TGF-β1 in matrix (faint yellow) were distributed in the islet β cell area (red) and surrounded islet β cells (top panels, scale bar 50 µm). High magnification is shown in bottom panels, scale bar 10 µm. Isotype-matched mouse IgG_1_ served as a negative control for TGF-β1 immunostaining in a serial pancreatic section. Representative images were obtained from five experiments. (B) TUNEL assay. The proteinase K-pretreated pancreatic slides were initially immunostained with TGF-β1 (yellow) and insulin (red) Abs, followed by TUNEL assay (green), and nuclear counterstaining with DAPI (blue). Scale bar 20 µm. Representative images were obtained from four experiments. (C) Percentage of apoptotic cells in subtypes of infiltrated leukocytes in pancreatic islets. Cryosections (8 µm thickness) of frozen pancreata from mCD4CD62L Treg-treated diabetic mice (n = 4) and control mice (n = 4) were initially detected with In Situ Cell Death Detection Kit (Roche), followed by immunostaining with different monoclonal Abs and imaging with a Zeiss LSM 510 META confocal microscope. Cryosections incubated with label solution without the TUNEL reaction mixture and/or isotype-matched IgG served as negative controls. Data represent mean±s.d. of five experiments.

TGF-β1 is one of the best characterized cytokines contributing to the induction of immune suppression and maintaining of self-tolerance [36]. To elucidate *de novo* molecular mechanism underlying the protection of islet β cells following treatment with mCD4CD62L Tregs, we determined TGF-β1 expression in pancreatic islets by immunohistochemistry in addition to plasma TGF-β1 measurement (**Figure 3F**). Results demonstrated that TGF-β1 was presented at higher level in pancreatic islets of mCD4CD62L Treg-treated diabetic mice compared with control CD4CD62L Treg-treated diabetic mice (**Figure 4A and B**). Staining of TGF-β1-positive cells showed two patterns: one was distributed among islet β cells, with average positive cell number of 14±9 cells/islet, and another was located around islet β cells. Importantly, we found that these surrounding TGF-β1-positive cells (negative for macrophage marker F4/80, but positive for dendritic cell marker CD11c [39], data not shown), along with their released TGF-β1 in the matrix (faint staining), formed a ring surrounding pancreatic islets (**Figure 4A**). This ring may protect newly-generated islets against attack by inducing apoptosis of auto-aggressive effector lymphocytes, as determined by terminal deoxynucleotidyl transferase dUTP nick end labeling (TUNEL) staining (**Figure 4B**, 58±23 TUNEL^+^ infiltrated leukocytes in mCD4CD62L Treg-treated group vs. 9±3 TUNEL^+^ infiltrated leukocytes in control CD4CD62L Treg-treated group, *p* = 0.02). To clarify which cell type became apoptotic, we performed double staining with different cell markers including CD4 for CD4^+^ T cells, CD8 for CD8^+^ T cells, B220 for B cells, and F4/80 for macrophages respectively in combination TUNEL staining. We found that treatment with mCD4CD62L Tregs increased the apoptosis of infiltrated T cells, B cells, and macrophages compared with control CD4CD62L Treg treatment (*p* = 0.0034, *p* = 0.024, *p* = 0.041, and *p* = 0.032 respectively). In comparison with the other three cell types however, CD4^+^ T cells showed a much higher percentage of apoptotic cells (**Figure 4C**). Thus, these data suggest that treatment with mCD4CD62L Tregs enhances expression of TGF-β1 in pancreatic islets that may contribute to local protection of newly-generated pancreatic islets from the re-destruction of autoreactive immune cells.

## Discussion

Millions of T1D patients worldwide must have daily insulin injections to survive. However, it is not a cure; it does not halt the persistent autoimmune response. Nor can it reliably prevent devastating complications such as neuronal and cardiovascular diseases, blindness, and kidney failure. This compelling need brings a sense of urgency to find a cure for T1D that can not only overcome the shortage of insulin-producing β-cells, but also halt the progression of autoimmunity [1], [5], [40]. Here we demonstrate that treatment with autologous mCD4CD62L Tregs can reverse established T1D, not only by controlling the autoimmunity but also by promoting β-cell regeneration leading to the restoration of euglycemia in a diabetic NOD mouse model. Thus, these findings provide a new approach for the treatment of T1D.

Tregs are fundamental in controlling immune responses and inducing self-tolerance. Functional defects of Tregs, not numerical, are essential for the development of T1D [10], [28]–[31]. In this study, we found that the frequency of Tregs such as CD4^+^CD25^+^ Treg, CD4^+^CD25^+^ Foxp3 Treg, and CD4^+^CD62L^+^ Treg failed to show significant differences at different stages of diabetes in comparison with pre-diabetic and/or non-diabetic NOD mice (data not shown). Additionally, treatment with control CD4CD62L Tregs could not reverse diabetes onset. Therefore, these data imply that the intrinsic functional defects of Tregs in NOD mice are consistent with previous reports [10], [28]–[30]. To date, little is known about how to correct the functional defects of Tregs. Based on our previous work [33], our current study demonstrated that multiple function-related genes have been markedly modulated in CD4^+^CD62L^+^ Tregs after coculture with CB-SC. Moreover, overt diabetic mice treated with the purified mCD4CD62L Tregs showed an elimination of hyperglycemia and a marked improvement in insulin production, which is correlated with a significant increase in β-cell mass and proliferation of β cells. Mechanistic studies demonstrated that control of diabetes was correlated with systemic immune alterations including restoration of Th1/Th2 cytokine balance in blood, as well as local regulations in pancreatic islets through a unique distributional pattern of TGF-β1 that may protect islet β cells against the infiltrated lymphocytes. These data have demonstrated human cord blood stem cells CB-SC can correct functional defects of CD4^+^CD62L^+^ Tregs and restore their therapeutic potential to treat T1D.

Our immunostaining results showed that recent onset of diabetic NOD mice possess a low amount of β-cell mass before treatment (>80% destroyed), with a low frequency of proliferating beta cells. Notably, treatment with mCD4CD62L Tregs can strongly stimulate the proliferation of β cells that were double positive for insulin and a cell proliferation nuclear antigen Ki67. This resulted in restoration of total β-cell mass and reconstitution of normal pancreatic islet architecture. It implies that mCD4CD62L Tregs function not only as an immune regulator to control autoimmune responses, but also as a pancreatic islet β-cell stimulator to promote β-cell regeneration. Therefore, both key issues (autoimmunity and shortage of insulin-producing cells) for recent onset of T1D have been overcome simultaneously by the treatment with mCD4CD62L Tregs. For late-stage T1D, it will be necessary to combine mCD4CD62L Tregs with stem cell-derived insulin-producing cells and/or other β-cell surrogates due to the almost complete disappearance of β cells in severe diabetics. Thus, early treatment with mCD4CD62L Tregs is more effective in reversing T1D.

Application of autologous cells is an attractive strategy for cell-based therapies as it eliminates the problems of immune rejection and shortage of suitable donors. However, it is crucial to use healthy allogeneic stem cells to modulate intrinsic functional defects of patient's Tregs due to the potential defects of patient's stem cells (un-published data). To generate a large-scale culture of allogeneic stem cells, we have identified and characterized a unique type of stem cell (CB-SC) from human umbilical cord blood using a simple isolation technique (blood mononuclear cells that attach to non-tissue cultured-treated Petri dishes [3]). In comparison with using bone marrow-derived mesenchymal stem cells (MSC) for immune modulation [34], [41], application of CB-SC possess several unique advantages including large resources of cord blood worldwide, no risk to the donor, easy to culture and expand *in vitro*, along with possession of embryonic characteristics. More specifically, CB-SC tightly adhere to culture dishes with a large rounded morphology and are resistant to regular detaching method (trypsin/EDTA), it is easy to collect suspending lymphocytes after co-culture with a minimum CB-SC contamination (<1%) [3], [33]. Thus, co-culture with CB-SC represents an ideal co-culture model to study the stem cell-related immune regulation. In current study, it is unfeasible to generate a large scale of stem cells from mouse cord blood due to the limited blood volume (about 10 µl/mouse). Therefore, we use CB-SC to co-culture with NOD lymphocytes and investigate the therapeutic potential of Tregs. During co-culture, CD4CD62L Tregs can be “educated” by the favorable microenvironment created by CB-SC through cell to cell contact and soluble mediators [33]. After co-culture with CB-SC, only floating lymphocytes were collected for purification of mCD4CD62L Tregs. Thus, these mCD4CD62L Tregs are the autologous therapeutic product.

In conclusion, we have developed a unique approach to reverse established type 1 diabetes by using human CB-SC to modulate autologous CD4CD62L Tregs. This treatment not only eliminated hyperglycemia and restored islet architecture via a marked increase in β-cell proliferation, but also diminished the autoimmunity through systemic immune alterations and local regulations in pancreatic islets. These autologous mCD4CD62L Tregs can be readily generated by co-culture with allogeneic CB-SC for therapeutic applications without concern for immune rejection. Our findings outline a new strategy for the prevention and treatment of diabetes and other autoimmune diseases.

## Materials and Methods

### Mice

Female NOD/LtJ mice, aged 5–6 weeks, were purchased from Jackson Laboratories (Bar Harbor, ME) and maintained under pathogen-free conditions at the University of Illinois at Chicago. Blood glucose levels were monitored using an Ascensia ELITE glucometer (Bayer Corporation, Elkhart, IN) between 9 and 11 A.M. under nonfasting conditions. Female diabetic NOD/LtJ mice (at 24–28 weeks of age) with spontaneously-developed autoimmune diabetes as confirmed by weight loss, polyuria, and nonfasting blood glucose levels >250 mg/dL for at least 2 consecutive days [9], [13], [42], [43], were used for treatment, according to a protocol approved by the Animal Care Committee (ACC) of University of Illinois at Chicago.

### Stem cell culture and co-culture with lymphocytes

Human umbilical cord blood (60–120 ml/unit/bag) was purchased from Life-Source Blood Services (Glenview, IL), which were derived from healthy donors. Application of cord blood for our researching does not need the ethical approval from the University and sign any agreements with donors due to their commercial availability. Human cord blood-derived stem cells (CB-SC) were generated as previously described [3]. In brief, cord blood mononuclear cells were plated in 150×15 mm Petri dishes (Becton Dickinson Labware, Franklin Lakes, NJ, not tissue culture-treated dishes) at 1×10^6^cells/ml, 25 ml/dish in RPMI 1640 medium supplemented with 7% fetal bovine serum (Invitrogen, Carlsbad, CA), and incubated at 37°C, in 8% CO_2_. After 2–3 weeks, CB-SC growing at 80–90% confluence were co-cultured with mouse lymphocytes after removing all unattached cord blood mononuclear cells. For co-culture, mouse lymphocytes were isolated from 6–8 week-old NOD mouse spleens [33] and plated onto CB-SC at a ratio 1∶10 of CB-SC∶lymphocytes in 150×15 mm Petri dishes containing 25 ml RPMI 1640 medium supplemented with 7% fetal bovine serum (Invitrogen), and incubated at 37°C in an incubator with 8% CO_2_. After co-culture for 2–4 days, the suspending lymphocytes were collected for experiments with a minimum CB-SC contamination (<1% of floating cells were positive for a CB-SC marker human leukocyte common antigen CD45). Because CB-SC tightly adhere to the culture dishes and exhibit large rounded morphology, it is easy to distinguish lymphocytes from CB-SC and to collect them. In control experiments, lymphocytes were cultured in identical growth conditions but without CB-SC.

### Flow analysis and cell sorting

Flow analysis and cell sorting were performed as previously described [33]. For flow analysis, cells were incubated with rat anti-mouse CD16 monoclonal antibody (eBioscience, San Diego, CA) diluted in medium containing 2.5% horse serum (Vector Laboratories) for 15 min at 4°C to block Fc receptor and to prevent non-specific staining. Cells were incubated with rat anti-mouse monoclonal antibodies (eBioscience), including Alex Fluor® 647-conjugated CD3, FITC- or phycoerythrin (PE)-conjugated CD4, FITC-conjugated CD25, and/or phycoerythrin-Cy7 (PE-Cy7)-conjugated CD62L for 45 min at 4°C and then washed with cold PBS prior to flow analysis. Isotype-matched rat anti-mouse IgG antibodies (eBioscience) served as negative control. After staining, cells were analyzed using a CyAn ADP (DakoCytomation). For intra-cellular cytokine staining, cells were initially stained for cell surface antigens (e.g., PE-conjugated CD4, FITC-conjugated CD25, and PE-Cy7-conjugated CD62L) and then prepared by using a BD Cytofix/Cytoperm Fixation/Permeabilization kit (BD Biosciences, San Jose, CA). Subsequently, cells were stained with different combinations of antibodies including FITC-conjugated IL-4, Alexa Fluor® 647-conjugated IL-10, Alexa Fluor® 647-conjugated IL-12, Pacific blue-conjugated IFN-γ (eBioscience), biotinylated anti-TGF-β1 Ab (Catalog number BAF240, R & D Systems, Minneapolis, MN). For TGF-β1 staining, cells were restained with strepavidin-conjugated FITC (Vector Laboratories). Alexa Fluor 647-conjugated anti-Foxp3 was purchased from eBioscience. For cell sorting to isolate different cell populations CB-SC-co-cultured, and control mouse lymphocytes, or freshly-isolated mouse splenocytes were initially incubated with CD16 Ab to block Fc receptor binding and then incubated with different combination of antibodies such as FITC-conjugated CD4 and PE-Cy7-conjugated CD62L for 45 min at 4°C and subjected to cell sorting using MoFlo (DakoCytomation). After confirming the purity of the population (>98%), CD4^+^CD62L^+^ Tregs were collected and used in different *in vitro* and *in vivo* experiments.

### Quantitative real time PCR array

Expression of different mRNAs was analyzed by quantitative real-time PCR. Total RNA was extracted using a Qiagen kit (Valencia, CA). First-strand cDNAs were synthesized from total RNA using QuantiTect Reverse Transcription kit according to the manufacturer's instructions (Qiangen, Valencia, CA). Real-time PCR was performed on each sample in triplicate using the ABI Prism 7900HT Fast Real-Time PCR System (Applied Biosystems, CA), under the following conditions: 95°C for 15 min, then 40 cycles of 95°C for 15 s, and 60°C for 60 s, using the validated gene-specific RT^2^ PCR Primer sets for each gene (SuperArray, Frederick, MD). Expression level of each gene, relative to β-actin as an internal control, was determined. For real-time PCR array, a mouse Th1-Th2-Th3 PCR array kit was used according to the manufacturer's instructions. The data were analyzed using a web-based PCR array data analysis software provided by the manufacturer (SuperArray).

### In vivo treatment protocol

To treat established diabetic NOD mice, spleen lymphocytes isolated from female NOD mice at 6–8 weeks of age were co-cultured with CB-SC as described above [33]. After co-culture for 2–4 days, floating lymphocytes were collected for cell sorting as described above. The purified CD4^+^CD62L^+^ Tregs (mCD4CD62L Tregs, 3×10^6^ cells) were administered intraperitoneally into overt diabetic NOD mice in 100 µl PBS/mouse (i.p., close to pancreas) for the first dose, followed by a second dose at 2 million cells in 100 µl PBS/mouse (i.p., close to pancreas) one week later. Diabetic mice injected with same volume of PBS served as one control. Because of a marked decrease in lymphocyte viability after *in vitro* culture in the absence of CB-SC, the sorted CD4^+^CD62L^+^ Tregs from freshly-isolated mouse spleen lymphocytes without co-culture with CB-SC ( control CD4CD62L Tregs) served as an additional control. Blood glucose levels and body weights were monitored twice a week until termination of the experiment. Three weeks after initiation of treatment, glucose tolerance testing was done as described below (n = 3 for each group). At seven weeks after treatment initiation, control mice were sacrificed for pathology due to severe hyperglycemia (>600 mg/dL) and loss of body weight (>20%). Diabetes-free mice following treatment with mCD4CD62L Tregs were also sacrificed for histological examinations. To measure insulin, blood samples were collected from the tail vein. Blood insulin level was measured using an ultrasensitive mouse insulin enzyme-linked immunosorbent assay (EIA) kit (Alpco Diagnostics, NH) following the manufacturer's protocols. The sensitivity of the assay is 0.019 ng/ml.

### Intraperitoneal glucose tolerance testing (IPGTT)

Mice were fasted overnight (12 h), weighed and injected intraperitoneally with a bolus of glucose (2 mg/g of body weight). Blood was then drawn from a tail vein at 0, 10, 20, 30, 45, 60, 90, and 120 min after glucose challenge. Glucose levels were measured from whole tail vein blood as described above.

### Immunohistochemistry and histology

Pancreata were fixed in 10% formaldehyde, processed, and embedded in paraffin. Serial sections were cut at 5 µm thickness. Immunostaining was performed as previously described with minor modifications [3]. To block non-specific staining, sections were incubated in a buffer containing 2.5% horse serum (Vector Laboratories) for 20 min at room temperature. Primary antibodies included guinea pig polyclonal anti-insulin Ab (DakoCytomation, Carpinteria, CA), mouse anti-glucagon mAb (Sigma), mouse anti-TGF-β1 mAb (Catalog number MAB240, 25% cross-reactivity with latent form of TGF-β1, no cross-reactivity with TGF-β2, <2% cross-reactivity with TGF-β3 and TGF-β5, R & D Systems), mouse anti-SMAD4 mAb (Santa Cruz Biotechnology, Santa Cruz, CA), rabbit anti-Ki67 mAb and rat anti-macrophage marker F4/80 mAb (Novus Biologicals, Littleton, CO), and hamster anti-mouse dendritic cell marker CD11c (BD Pharmingen). Second Abs included Texas red-conjugated AffiniPure donkey anti-guinea pig IgG, rhodamine-conjugated AffiniPure donkey anti-rabbit IgG, AMCA AffiniPure Donkey Anti-Rabbit IgG, FITC-conjugated AffiniPure donkey anti-mouse IgG, and Cy5-conjugated AffiniPure donkey anti-mouse IgG, AMCA AffiniPure Donkey Anti-armenian hamster IgG, and Cy5-conjugated AffiniPure donkey anti-rat IgG (Jackson ImmunoResearch Laboratories, West Grove, PA). For non-fluorescence staining, after incubation with primary antibodies, cells were stained with an ABC kit (Vector Laboratories, Burlingame, CA). Biotinylated horse anti-rabbit Ab and biotinylated goat anti-guinea Ab were purchased from Vector Laboratories (Burlingame, CA). For isotype-matched controls, mouse IgG_1κ_ was purchased from BD Biosciences, guinea pig serum and rabbit IgG from Santa Cruz Biotechnology. For pancreatic slides, we counterstained with hematoxylin (Sigma) after immunostaining. For every experiment, isotype-matched antibodies were used as negative controls. Cells were photographed with a Zeiss Axiocam Color Camera using Zeiss Axioskop Histology/Digital Fluorescence microscope for HRP-immunostaining images, with Zeiss LSM 510 META confocal microscope for fluorescence images.

To compare total β-cell mass after immunostaining with insulin Ab, β-cell mass was measured and calculated by point-counting morphometric analysis [35] using Image J software, download from the NIH website (http://rsbweb.nih.gov/ij/).

To score insulitis, pancreatic sections from each experimental group were stained with hematoxylin and eosin (H&E staining, Sigma). At least 50 islets from 200 serial sections of each pancreas were examined to evaluate the degree of leukocyte infiltration. Insulitis was graded into five categories based on the extent of intra-islet infiltration of leukocytes: no insulitis (no infiltration), mild insulitis (<25% infiltration), moderate insulitis (25%∼50% infiltration), severe insulitis (50%∼75% infiltrations), and profound insulitis (>75% infiltration).

To determine apoptosis of infiltrated leukocytes, in situ cell death detection kit (fluorescein) (Roche Applied Science, Indianapolis, IN) was applied and performed using the manufacturer's recommended protocol. Cryosections (8 µm thickness) of frozen pancreata from mCD4CD62L Treg-treated diabetic mice and control group were prepared by using Microtome Cryostat HM 500 OM (Microm International GmbH). To determine which cell type became apoptotic, we use different markers including PE-conjugated CD4 mAb for CD4^+^ T cells, PE-conjugated CD8 mAb for CD8^+^ T cells, PE-conjugated B220 mAb for B cells, and rat anti-mouse F4/80 mAb for macrophages respectively in combination with TUNEL staining. The mAbs to CD4, CD8 and B220 were from eBioscience. Cryosections were initially detected with In Situ Cell Death Detection Kit (Roche), followed by immunostaining with different monoclonal Abs and imaging with a Zeiss LSM 510 META confocal microscope. After double staining, positive cells were quantified directly on the confocal microscope and/or on images. Cryosections incubated with label solution without TUNEL reaction mixture and/or isotype-matched IgG served as negative controls.

### Cytokine assays

Cytokine levels in mouse plasma were quantified using commercial ELISA kits following manufacturer's instructions. We purchased mouse IFN-γ ELISA kit from Biolegend Inc.(San Diego, CA), mouse IL-4 and IL-10 ELISA kits from Assay Designs (Ann Arbor, MI), and TGF-β1 ELISA kit from Promega (Madison, WI).

### Statistical analysis

Statistical analyses of data were performed by the two-tailed Student's t-test to determine statistical significance. Values are given as mean±SD (standard deviation).

## Supporting Information

Table S1 Modulation of Th1-Th2-Th3-related gene expressions in CD4^+^CD62L^+^ Tregs after CB-SC co-culture. Mouse spleen lymphocytes were isolated from female NOD mice (aged 6–8 weeks) and then co-cultured with CB-SC for 2 days at a ratio 1∶10 of CB-SC∶lymphocytes. Subsequently, the total lymphocytes were harvested for cell sorting. Lymphocytes cultured in absence of CB-SC served as control. Total RNAs were extracted from the purified CD4^+^CD62L^+^ Tregs using a Qiagen kit (Valencia, CA). First-strand cDNAs were synthesized RNA using QuantiTect Reverse Transcription kit (Qiangen). For real-time PCR array, mouse Th1-Th2-Th3 PCR array kits were used according to the manufacturer's instructions: 95°C for 15 min, then 40 cycles of 95°C for 15 s, and 60°C for 60 s, followed by a web-based PCR array data analysis provided by the manufacturer (SuperArray). Relative expression level of each gene was corrected for that of the housekeeping gene β-actin as an internal control. Data represent one of three independent experiments that gave similar results. Fold change of expression in mCD4CD62L Tregs compared with control CD4CD62L Tregs is shown.
(0.03 MB DOC)

## References

[1] Trucco M (2005). Regeneration of the pancreatic beta cell.. J Clin Invest.

[2] Zhao Y, Glesne D, Huberman E (2003). A human peripheral blood monocyte-derived subset acts as pluripotent stem cells.. Proc Natl Acad Sci U S A.

[3] Zhao Y, Wang H, Mazzone T (2006). Identification of stem cells from human umbilical cord blood with embryonic and hematopoietic characteristics.. Exp Cell Res.

[4] Ricordi C, Hering BJ, Shapiro AM (2008). Beta-cell transplantation for diabetes therapy.. Lancet.

[5] Ablamunits V, Sherry NA, Kushner JA, Herold KC (2007). Autoimmunity and beta cell regeneration in mouse and human type 1 diabetes: the peace is not enough.. Ann N Y Acad Sci.

[6] Bonner-Weir S, Weir GC (2005). New sources of pancreatic beta-cells.. Nat Biotechnol.

[7] Zhou Q, Brown J, Kanarek A, Rajagopal J, Melton DA (2008). In vivo reprogramming of adult pancreatic exocrine cells to beta-cells.. Nature.

[8] Bluestone JA, Tang Q, Sedwick CE (2008). T Regulatory Cells in Autoimmune Diabetes: Past Challenges, Future Prospects.. J Clin Immunol.

[9] Bresson D, Togher L, Rodrigo E, Chen Y, Bluestone JA (2006). Anti-CD3 and nasal proinsulin combination therapy enhances remission from recent-onset autoimmune diabetes by inducing Tregs.. J Clin Invest.

[10] Chatenoud L, Bach JF (2005). Resetting the functional capacity of regulatory T cells: a novel immunotherapeutic strategy to promote immune tolerance.. Expert Opin Biol Ther.

[11] Chatenoud L, Bach JF (2005). Regulatory T cells in the control of autoimmune diabetes: the case of the NOD mouse.. Int Rev Immunol.

[12] Roncarolo MG, Battaglia M (2007). Regulatory T-cell immunotherapy for tolerance to self antigens and alloantigens in humans.. Nat Rev Immunol.

[13] Tarbell KV, Petit L, Zuo X, Toy P, Luo X (2007). Dendritic cell-expanded, islet-specific CD4+CD25+CD62L+ regulatory T cells restore normoglycemia in diabetic NOD mice.. J Exp Med.

[14] You S, Slehoffer G, Barriot S, Bach JF, Chatenoud L (2004). Unique role of CD4+CD62L+ regulatory T cells in the control of autoimmune diabetes in T cell receptor transgenic mice.. Proc Natl Acad Sci U S A.

[15] Li Q, Xu B, Michie SA, Rubins KH, Schreriber RD (2008). Interferon-alpha initiates type 1 diabetes in nonobese diabetic mice.. Proc Natl Acad Sci U S A.

[16] Schneider A, Rieck M, Sanda S, Pihoker C, Greenbaum C (2008). The effector T cells of diabetic subjects are resistant to regulation via CD4+FOXP3+ regulatory T cells.. J Immunol.

[17] Tang Q, Adams JY, Penaranda C, Melli K, Piaggio E (2008). Central role of defective interleukin-2 production in the triggering of islet autoimmune destruction.. Immunity.

[18] Zehn D, Bevan MJ (2006). T cells with low avidity for a tissue-restricted antigen routinely evade central and peripheral tolerance and cause autoimmunity.. Immunity.

[19] Allen JS, Pang K, Skowera A, Ellis R, Rackham C (2008). Plasmacytoid dendritic cells are proportionally expanded at diagnosis of Type 1 diabetes and enhance islet autoantigen presentation to T cells through immune complex capture.. Diabetes.

[20] Huang Y, Fugier-Vivier IJ, Miller T, Elliott MJ, Xu H (2008). Plasmacytoid precursor dendritic cells from NOD mice exhibit impaired function: are they a component of diabetes pathogenesis?. Diabetes.

[21] Jin Y, Chen X, Podolsky R, Hopkins D, Makala LH (2008). APC dysfunction is correlated with defective suppression of T cell proliferation in human type 1 diabetes.. Clin Immunol.

[22] Marleau AM, Summers KL, Singh B (2008). Differential contributions of APC subsets to T cell activation in nonobese diabetic mice.. J Immunol.

[23] Summers KL, Marleau AM, Mahon JL, McManus R, Hramiak I (2006). Reduced IFN-alpha secretion by blood dendritic cells in human diabetes.. Clin Immunol.

[24] Kukreja A, Cost G, Marker J, Zhang C, Sun Z (2002). Multiple immuno-regulatory defects in type-1 diabetes.. J Clin Invest.

[25] Pop SM, Wong CP, Culton DA, Clarke SH, Tisch R (2005). Single cell analysis shows decreasing FoxP3 and TGFbeta1 coexpressing CD4+CD25+ regulatory T cells during autoimmune diabetes.. J Exp Med.

[26] You S, Belghith M, Cobbold S, Alyanakian MA, Gouarin C (2005). Autoimmune diabetes onset results from qualitative rather than quantitative age-dependent changes in pathogenic T-cells.. Diabetes.

[27] Bayry J, Lacroix-Desmazes S, Dasgupta S, Kazatchkine MD, Kaveri SV (2008). Efficacy of regulatory T-cell immunotherapy: are inflammatory cytokines key determinants?. Nat Rev Immunol.

[28] Brusko T, Wasserfall C, McGrail K, Schatz R, Viener HL (2007). No alterations in the frequency of FOXP3+ regulatory T-cells in type 1 diabetes.. Diabetes.

[29] Brusko TM, Wasserfall CH, Clare-Salzler MJ, Schatz DA, Atkinson MA (2005). Functional defects and the influence of age on the frequency of CD4+CD25+ T-cells in type 1 diabetes.. Diabetes.

[30] Gombert JM, Herbelin A, Tancrede-Bohin E, Dy M, Chatenoud L (1996). Early defect of immunoregulatory T cells in autoimmune diabetes.. C R Acad Sci III.

[31] Lindley S, Dayan CM, Bishop A, Roep BO, Peakman M (2005). Defective suppressor function in CD4(+)CD25(+) T-cells from patients with type 1 diabetes.. Diabetes.

[32] Tritt M, Sgouroudis E, d'Hennezel E, Albanese A, Piccirillo CA (2008). Functional waning of naturally occurring CD4+ regulatory T-cells contributes to the onset of autoimmune diabetes.. Diabetes.

[33] Zhao Y, Huang Z, Qi M, Lazzarini P, Mazzone T (2007). Immune regulation of T lymphocyte by a newly characterized human umbilical cord blood stem cell.. Immunol Lett.

[34] Abdi R, Fiorina P, Adra CN, Atkinson M, Sayegh MH (2008). Immunomodulation by mesenchymal stem cells: a potential therapeutic strategy for type 1 diabetes.. Diabetes.

[35] Meier JJ, Lin JC, Butler AE, Galasso R, Martinez DS (2006). Direct evidence of attempted beta cell regeneration in an 89-year-old patient with recent-onset type 1 diabetes.. Diabetologia.

[36] Li MO, Flavell RA (2008). TGF-beta: a master of all T cell trades.. Cell.

[37] Roncarolo MG, Battaglia M, Gregori S (2003). The role of interleukin 10 in the control of autoimmunity.. J Autoimmun.

[38] Wan YY, Flavell RA (2006). The roles for cytokines in the generation and maintenance of regulatory T cells.. Immunol Rev.

[39] Perruche S, Zhang P, Liu Y, Saas P, Bluestone JA (2008). CD3-specific antibody-induced immune tolerance involves transforming growth factor-beta from phagocytes digesting apoptotic T cells.. Nat Med.

[40] Staeva-Vieira T, Peakman M, von HM (2007). Translational mini-review series on type 1 diabetes: Immune-based therapeutic approaches for type 1 diabetes.. Clin Exp Immunol.

[41] Nauta AJ, Fibbe WE (2007). Immunomodulatory properties of mesenchymal stromal cells.. Blood.

[42] Bour-Jordan H, Salomon BL, Thompson HL, Santos R, Abbas AK (2007). Constitutive expression of B7-1 on B cells uncovers autoimmunity toward the B cell compartment in the nonobese diabetic mouse.. J Immunol.

[43] Goudy KS, Burkhardt BR, Wasserfall C, Song S, Campbell-Thompson ML (2003). Systemic overexpression of IL-10 induces CD4+CD25+ cell populations in vivo and ameliorates type 1 diabetes in nonobese diabetic mice in a dose-dependent fashion.. J Immunol.

