# Supplementary material for: Human Cord Blood Stem Cell-Modulated Regulatory T Lymphocytes Reverse the Autoimmune-Caused Type 1 Diabetes in Nonobese Diabetic (NOD) Mice
Source: PLoS One. 2009 Jan 19;4(1):e4226. doi: 10.1371/journal.pone.0004226 (PMC2627485; doi:10.1371/journal.pone.0004226)
Supplement: Table S1 — Modulation of Th1-Th2-Th3-related gene expressions in CD4+CD62L+ Tregs after CB-SC co-culture. Mouse spleen lymphocytes were isolated from female NOD mice (aged 6–8 weeks) and then co-cultured with CB-SC for 2 days at a ratio 1∶10 of CB-SC∶lymphocytes. Subsequently, the total lymphocytes were harvested for cell sorting. Lymphocytes cultured in absence of CB-SC served as control. Total RNAs were extracted from the purified CD4+CD62L+ Tregs using a Qiagen kit (Valencia, CA). First-strand cDNAs were synthesized RNA using QuantiTect Reverse Transcription kit (Qiangen). For real-time PCR array, mouse Th1-Th2-Th3 PCR array kits were used according to the manufacturer's instructions: 95°C for 15 min, then 40 cycles of 95°C for 15 s, and 60°C for 60 s, followed by a web-based PCR array data analysis provided by the manufacturer (SuperArray). Relative expression level of each gene was corrected for that of the housekeeping gene β-actin as an internal control. Data represent one of three independent experiments that gave similar results. Fold change of expression in mCD4CD62L Tregs compared with control CD4CD62L Tregs is shown. (0.03 MB DOC) [file pone.0004226.s001.doc]

**Table S1. Modulation of Th1-Th2-Th3-related gene expressions in CD4^+^CD62L^+^ Tregs after CB-SC co-culture.**

**Note:** Mouse spleen lymphocytes were isolated from female NOD mice (aged 6-8 weeks) and then co-cultured with CB-SC for 2 days at a ratio 1:10 of CB-SC : lymphocytes. Subsequently, the total lymphocytes were harvested for cell sorting. Lymphocytes cultured in absence of CB-SC served as control. Total RNAs were extracted from the purified CD4^+^CD62L^+^ Tregs using a Qiagen kit (Valencia, CA). First-strand cDNAs were synthesized RNA using QuantiTect Reverse Transcription kit (Qiangen). For real-time PCR array, mouse Th1-Th2-Th3 PCR array kits were used according to the manufacturer's instructions: 95 °C for 15 min, then 40 cycles of 95°C for 15 s, and 60 °C for 60 s, followed by a web-based PCR array data analysis provided by the manufacturer (SuperArray). Relative expression level of each gene was corrected for that of the housekeeping gene β-actin as an internal control. Data represent one of three independent experiments that gave similar results. Fold change of expression in mCD4CD62L Tregs compared with control CD4CD62L Tregs is shown.
